# Exposure-Based Virtual Reality Interventions for Anxiety Disorders: A Systematic Review and Thematic Synthesis of Patient and Therapist Perspectives

**DOI:** 10.1192/bjo.2026.11156

**Published:** 2026-06-30

**Authors:** Laura Chapman, Peter Thurlow, Lucy Biddle, Helen Bould

**Affiliations:** 1 University of Bristol, Bristol, United Kingdom; 2 Avon and Wiltshire Mental Health Partnership NHS Trust, Bristol, United Kingdom; 3 Gloucestershire Health and Care NHS Foundation Trust, Gloucester, United Kingdom

## Abstract

**Aims:**

Immersive virtual reality (VR) is increasingly being explored as a potential modality for the assessment and treatment of a range of mental health conditions, including anxiety disorders. To date, many of the VR interventions that have been developed for anxiety disorders draw upon the principles of exposure therapy, and a growing body of evidence has begun to demonstrate their efficacy. However, little is known about the ways in which the individuals for whom these interventions are designed perceive and experience them, despite the importance of understanding both patient and practitioner perspectives to enable effective intervention development and evaluation. The aim of this systematic review was to identify and synthesise the existing qualitative evidence-base in relation to exposure-based immersive VR interventions for the treatment of anxiety disorders.

**Methods:**

We systematically searched PubMed, Scopus, Medline, Embase, PsychINFO, OVID interface, MedArXiv and PsyArXiv for qualitative and mixed-methods studies reporting patient and therapist perspectives, experiences and recommendations relevant to the focus of our review. In total, 9714 abstracts were screened and 954 full-text manuscripts were retrieved, with 12 studies meeting the review inclusion criteria. The qualitative results sections of articles were coded inductively line-by-line, and the data was synthesised using thematic synthesis.

**Results:**

Twelve descriptive themes and four analytical themes were generated through the analysis. The analytical themes were: 1. *Contingent experiences of* *anxiety* (facilitators of and barriers to experiencing symptoms of anxiety during VR exposure); 2. *Learning beyond* *exposure* (the ways in which participants experienced benefits from VR exposure that extended beyond the intended mechanisms of change underpinning exposure therapy approaches); 3. “*Somewhere between being there and not being there*” (the nature of an anxiety-provoking ‘reality’ that is virtual, and how experiences of the virtual world relate to experiences of anxiety in real life); and 4. *The patient, the therapist, and the Head-Mounted Device* (how VR exposure-based interventions might best be positioned and delivered in the context of psychotherapy, and how VR might present both benefits and barriers for patients, therapists, and for the therapeutic relationship).

**Conclusion:**

This synthesis of the perspectives, experiences and recommendations of patients and therapists in relation to exposure-based VR interventions for anxiety disorders has a range of implications, for both practice and research. These include a number of recommendations for the design, delivery and evaluation of VR interventions for anxiety disorders, ensuring they better meet the needs of the people who use them.

